# Protective and Anti-Inflammatory Effect of Novel Formulation Based on High and Low Molecular Weight Hyaluronic Acid and *Salvia haenkei*

**DOI:** 10.3390/ijms26031310

**Published:** 2025-02-04

**Authors:** Maria d’Agostino, Andrea Maria Giori, Valentina Vassallo, Chiara Schiraldi, Antonella D’Agostino

**Affiliations:** 1 Department of Experimental Medicine, Section of Biotechnology, Medical Histology and Molecular Biology, University of Campania “Luigi Vanvitelli”, via L. De Crecchio 7, 80138 Naples, Italy; maria.dagostino@unicampania.it (M.d.); valentina.vassallo@unicampania.it (V.V.); 2 R&D Department, IBSA Farmaceutici Italia, 26900 Lodi, Italy; 3 Department of Life Sciences, Health and Health Professions, Link Campus University, 00165 Rome, Italy

**Keywords:** *Salvia haenkei*, hyaluronan, keratinocytes, dehydration, wound healing, lipopolysaccharides, anti-inflammatory effects, cytokines, 3D-FT skin

## Abstract

*Salvia haenkei* (SH-Haenkenium^®^), a native plant of Bolivia, is known as strong inhibitor of senescence and recently exploited in wound healing and for its potential anti-inflammatory properties. Hyaluronan at high and low molecular weight (HCC), explored in diverse cell models, and recently used in clinical practice, showed beneficial effects in dermo aesthetic and regenerative injective treatments. In this research work a novel formulation based on HCC coupled SH was tested for its potentiality in counteracting dermal injury. In vitro wound healing has been used to demonstrate HCC + SH capacity to improve keratinocytes migration respects the sole HCC, supported also by positive modulation of remodeling and integrity biomarkers. In addition, an in vitro dehydration test showed its ability to defend the skin from dryness. Moreover, an in vitro inflammation model (with lipopolysaccharides derived from *E. coli*) was used to assess molecular fingerprint of the pathological model and compare the cell response after treatments. Inflammatory biomarkers (e.g., KRT6, TLR-4 and NF-κB) and specific cytokines (e.g., IL-6, IL-22, IL-23) proved the effect of HCC + SH, in reducing inflammatory mediators. A more complex model, 3D-FT skin, was used to better resemble an in vivo condition, and confirmed the efficacy of novel formulations to counteract inflammation. All results trigger the interest in the novel formulation based on SH extract and hyaluronan complexes for its potential efficacy as natural anti-inflammatory agent for damaged skin, for its healing and regenerative properties.

## 1. Introduction

Scientific community, over the years, has been studying the complex mechanism of tissue damage/regeneration using natural polymers eventually modified that may assist or even prompt the spontaneous reparation to obtain a healthy tissue. Alternatively, other studies aim to hamper or reduce inflammation status associated to dermatological pathologies. Hyaluronic acid (HA) is one of the components of extracellular matrix (ECM), widely exploited in tissue diseases having a positive modulation in skin healing. The use of different sizes of HA samples alone or in combination is widely exploited in scientific research especially in cosmeceuticals and generally in biomedical applications [1,2,3]. In fact, a formulation based on high- and low molecular weight HA, known as hybrid cooperative complexes (HCC) was widely exploited for several applications as topical cream, injective preparation currently used in dermal treatment [4,5,6]. Indeed, HA with an average of 1000 kDa (HHA) generally protects tissues, shows immunosuppressive functions, and improves skin hydration [7]. Low molecular weight HA (LHA), ranging from 10 to 250 kDa usually triggers mild inflammatory and immune responses, and it can penetrate more easily the skin respect HHA [8]. Other studies focused on the role of different molecular weight of HA in pathologies associated to common chronic skin diseases, as atopic dermatitis (AD), proving that, by means of phosphatidylinositol 3-kinases (PI3K)/Protein kinase B (Akt)pathway, the reduction of inflammation response in macrophages, namely RAW 264.7 cells [9,10]. On the other side, the use of natural compound as hydroalcoholic extract of *Salvia haenkei* (SH-Haenkenium^®^), a Bolivian plant, was recently investigated for its antiaging properties [11]. These studies proved SH acts as a scavenger of ROS, promotes junction’s reinforcement and increases keratinocytes (HaCaT) cell migration [12]. SH was also evaluated, and especially coupled to hyaluronan, on atopic dermatitis (AD) lesions showing to protect skin integrity thus significantly reducing its severity [13]. Hence, the present research work aims to combine an HA hybrid complexes with SH (HCC + SH) to evaluate ability to prompt tissue repair and, in addition, to investigate their anti-inflammatory efficacy, with specific interest in an in vitro psoriatic model. Psoriasis (PS) is mainly characterized by a disorder in the immune response with successive scaly and dryness. This pathological condition is mainly due to an abnormal proliferation of keratinocytes and persistent inflammation both in epidermis and dermis [14,15]. Generally, in vivo immune cells secrete cytokines that lead to keratinocytes hyperproliferation. Hence, up to date strategies widely exploited for psoriasis treatment focus on attenuating HaCaT hyperproliferation and/or persistent inflammation [16]. A recent study reported the potency of natural compounds, like genestin and daphnetin (Dap) in reducing specific psoriatic genes expression and cytokines both in vitro and in vivo models. Particularly, studies on Dap effect, proved that the inhibition of proliferation/inflammation in HaCaT occurred through the inactivation of NF-κB signaling pathway and significantly reduced some of PS key markers: IL-6 and IL-8 genes [17,18]. Moreover, several studies also demonstrated the efficacy of HCC to repair damaged skin in psoriasis [19] or acne scars [20,21] demonstrating the pivotal role of HCCs in regenerative medicine. In this research work, then, we evaluated the efficacy of HCC + SH suitable for skin regeneration in wound healing models by time lapse experiments and remodeling specific biomarkers of regeneration as elastin, integrins and aquaporins as evaluated elsewhere [22]. Furthermore, an in vitro inflammation, commonly used in psoriatic model, specifically HaCaT treated with LPS, was investigated to obtain “inflamed keratinocytes” and to study the potential reduction of inflammation status in presence of novel formulations based on HCC and SH. Ultimately, this study about the in vitro inflammation was implemented using 3D skin model for better mimic in vivo models and to obtain results more closely to real pathologies associated to dermal repair, especially for psoriasis diseases.

## 2. Results

### 2.1. Protective Effect Against Dehydration

**Figure 1:**
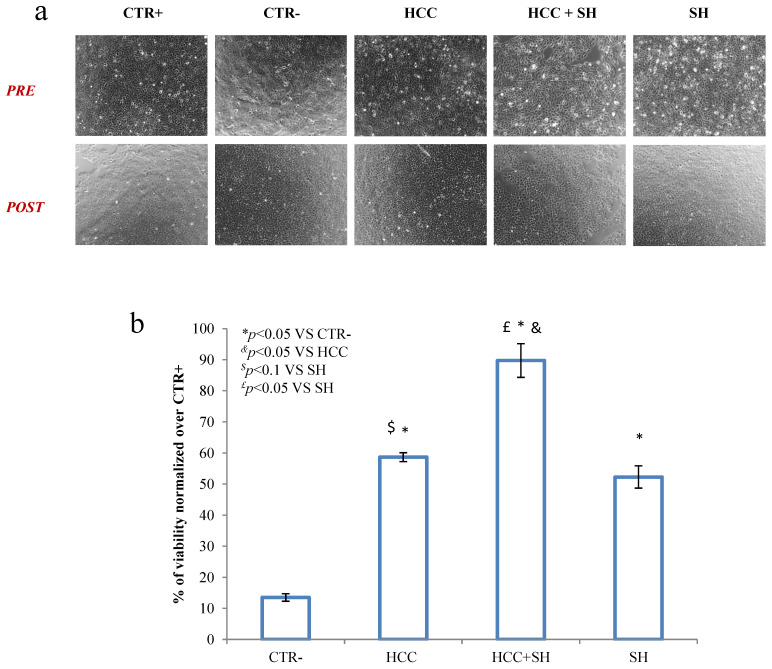
(**a**) Representative micrographs pictures of HaCaT before and after dehydration test. (**b**) Viability assay by MTT assay. *, & and £ stand for the statistical analyses output *p* < 0.05, $ stand for the statistical analyses output *p* < 0.1; area is picked by the software.

To evaluate formulation’s ability to counteract skin from dehydration status, HaCaT cell lines at 70% confluence and were observed prior and after dehydration (Figure 1a). Viability results (Figure 1b) normalized respect to the untreated/no dehydrated control (CTR+), confirmed the action of HCC + SH against this stressful condition, evident from cellular activity, when also compared to the CTR+. In more detail, all substances showed a beneficial effect when compared to the untreated/dehydrated (CTR−). In terms of viability HCC + SH revealed an improvement, in terms of action, compared to the formulations taken individually, but no significantly synergistic effect existed between the two treatments (HCC to SH).

### 2.2. Effect of HCC + SH in Wound Closure and in Remodeling Biomarker Evaluation

**Figure 2:**
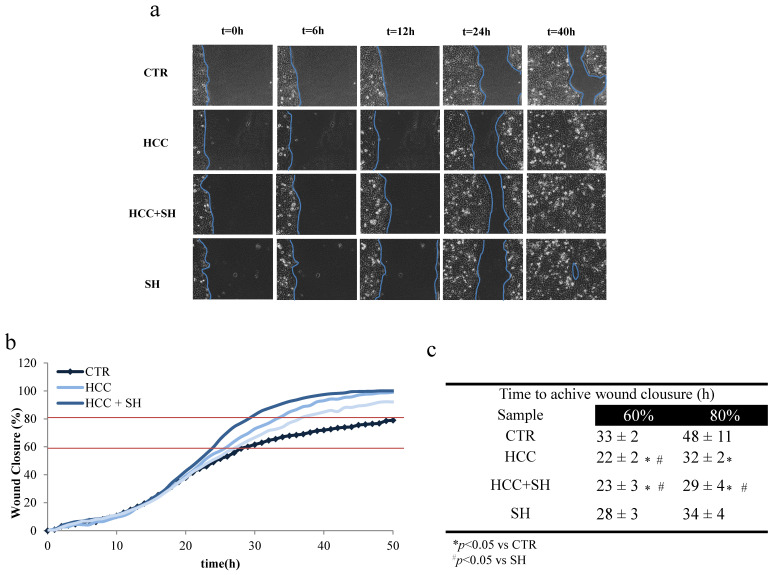
Wound healing assay (**a**) Representative pictures of HaCaT monolayer after the scratch and in time course of experiment recovered by time lapse software (OKO-vision 2009, 4.3 software). (**b**) Quantitative analysis of wound closure in the time. (**c**) Time to achieve 60% and 80% of wound closure in the CTR and in presence of HCC, HCC + SH and SH. * and # stand for the statistical analyses output *p* < 0.05; area is picked by the software.

The new formulations were used on scratched HaCaT monolayer, to evaluate whether they were able to promote tissue regeneration. The images panel (Figure 2a) provides a qualitative and direct model of the process. Figure 2b showed, in more detail, the closure processes and the effectiveness of all treatments in accelerating the repair process with respect to untreated/scratched control (CTR) cells. HCC + SH reached 80% wound closure on average earlier than the other treatments (Figure 2c).

**Figure 3:**
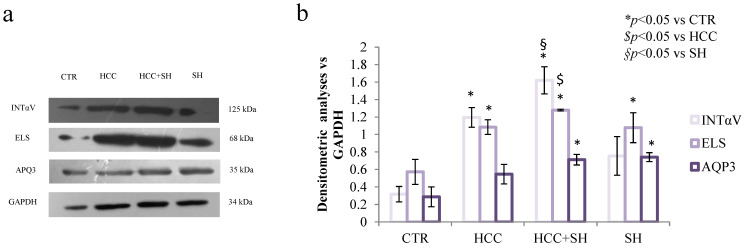
(**a**) Western blot analysis of INTαV, ELS and AQP3 in scratched HaCaT monolayer at 72 h in the CTR and in presence of different treatments. (**b**) The expression of each protein was normalized in respect to GAPDH used as housekeeping internal control. *, $ and § stand for the statistical analyses output *p* < 0.05.

At the end of experiment the intra-cellular expression levels of well-recognized remodeling, adhesion and cell integrity biomarkers were also analyzed by western blotting (respectively, Elastin, integrin αV and acquaporine-3). As clear in Figure 3, all treatments modulated positively in a statistical manner the production of considered biomarkers with respect to CTR. In particular, HCC and HCC + SH were able to enhance INTαV, 4 and 5-fold respectively to CTR. Similarly, the increase of ELS and AQP3 was 2-fold both for HCC and HCC + SH respect to CTR. Interestingly, an appreciable fold increase (~2.7) in presence of SH for INTαV. SH treatments improved ELS and AQP3 expression but to a lesser degree (Supplementary Materials Figure S1).

### 2.3. LPS-Induced HaCaT Cell Model: Effect of HCC and HCC + SH

#### Gene Expression

**Figure 4:**
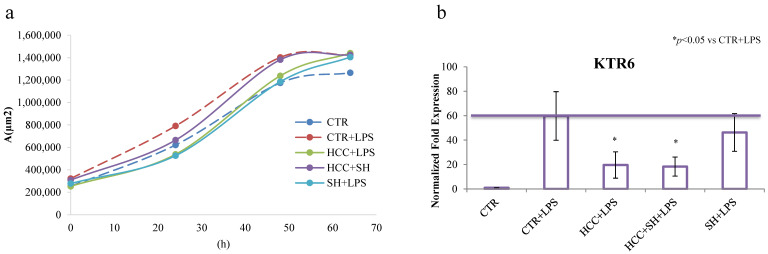

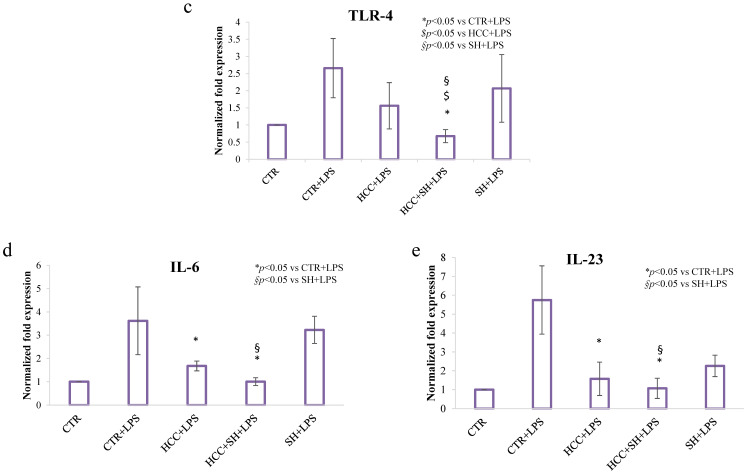
(**a**) HaCaT proliferation in presence of LPS stimulation performed by time lapse measuring the area occupied from the cells in the time. (**b**–**e**) qRT-PCR relative to KRT6, TLR-4, IL-6, IL-23 expression in the CTR and in presence of different treatments at 24 h. *, $ and § stand for the statistical analyses output *p* < 0.05.

In order to understand if the LPS induced HaCaT led to inflamed cells similar to psoriatic model, KRT6, considered as a hallmark of in vitro psoriatic model, was evaluated after 24 h of LPS stimulation [17]. qRT-PCR results showed that LPS stimulation significantly increased KRT6 mRNA levels that were instead statistically reduced by HCC and HCC + SH treatments (3-fold respect to CTR + LPS) (Figure 4a). At the same time, specific psoriatic/inflammatory markers, TLR-4, IL-6 and IL-23A, were also evaluated. Generally, all the treatments attenuated LPS-induced upregulation of those inflammatory markers without statistical significance, except for HCC + SH that reduced TLR-4, IL-6 and IL-23A, of about 3.8, 4 and 6-fold respectively compared to stimulated CTR (Figure 4c–e). Even if not to the same extent than the previous formulation, also HCC showed an anti-inflammatory action with TLR-4, IL-6 and IL-23A, 1.8, 2 and 4-fold lower than stimulated CTR respectively.

### 2.4. Protein Expression

**Figure 5:**
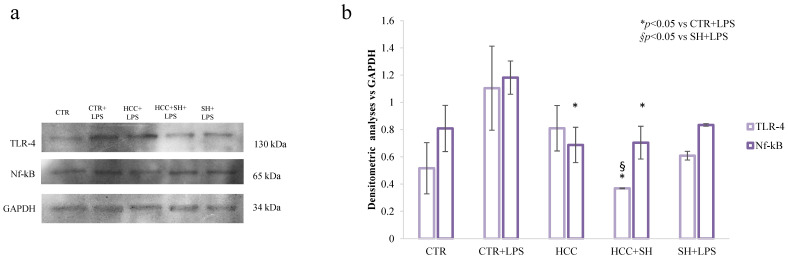
Western blot analysis in inflamed HaCaT cell at 72 h. GAPDH was used as the loading control. (**a**) Biomarkers involved in inflammation process. (**b**) The expression of each protein was normalized in respect to GAPDH used as housekeeping internal control. * and § stand for the statistical analyses output *p* < 0.05.

Furthermore, the quantification of TLR-4 and NF-κB proteins were assessed by western blot assay. The relative expressions revealed the reduction of TLR-4 and NF-κB expression, by all the treatments, compared to the CTR stimulated with LPS. In detail, TLR-4 expression was strongly diminished in HCC + SH treatment respects the single components, especially compared to sole SH (Figure 5). Regarding NF-κB expression, there was a less remarkable difference between HCC and HCC + SH samples (Supplementary Materials Figure S2).

### 2.5. Inflammatory Cytokines Quantification

**Figure 6:**
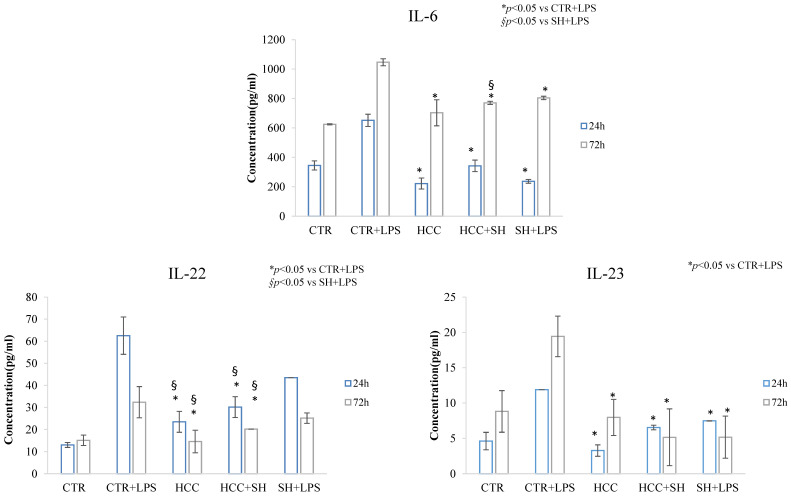
IL-6, IL-22, IL-23 cytokines quantification by ELISA assay after 24 h and 72 h of LPS stimulation. * and § stand for the statistical analyses output *p* < 0.05.

The inflammation and its reduction in presence of treatments were evaluated through cytokine production (IL-6, IL-22 and IL-23A) in stimulated LPS cells proliferation after 24 h and 72 h (Figure 6). All the treatments decreased significantly the cytokine level with respect to LPS stimulated CTR except for IL-22 in presence of SH at 24 h. For IL-22 (Figure 6), a significant value for HCC and HCC + SH respect to SH have been revealed at both the time. Regarding IL-6 (Figure 6), we found a less remarkable difference between CTR + LPS and the LPS stimulated cells pretreated with formulations, but these data was in accordance with literature [23].

### 2.6. LPS-Induced 3D Full-Thickness Skin (FT-Skin) Model: Effect of HCC and HCC + SH

#### 2.6.1. Gene Expression

**Figure 7:**
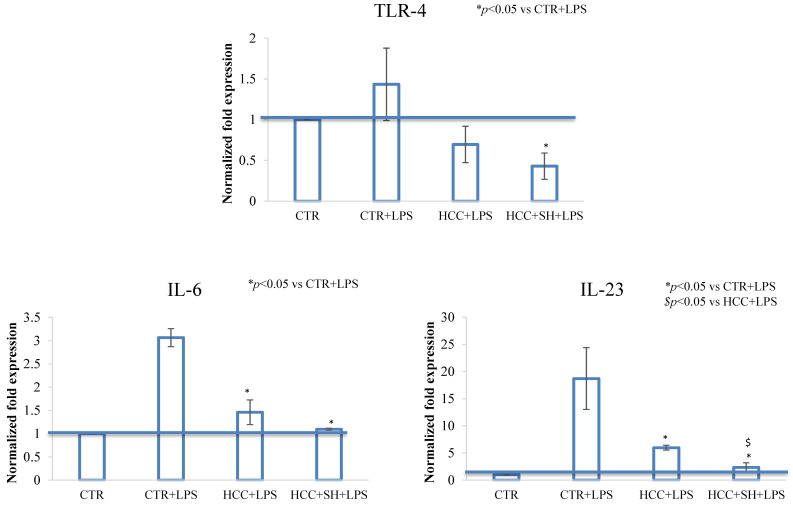
qRT-PCR of 3D-skin, relative to TLR-4, IL-6, IL-23 expression in the CTR and in presence of different treatments at 72 h. Blu lines indicated the CTR level. Values are calculated as mean ± SD of three different experiments and are expressed as mRNA normalized fold increase respect to CTR. * and $ stand for the statistical analyses output *p* < 0.05.

A final set of experiments were run using a 3D Full Thickness (FT) skin model, on the two most functional formulations, namely HCC and HCC + SH. Gene expression analyses on TLR-4, IL-6, IL-23 obtained through qRT-PCR, are presented in Figure 7, HCC and HCC + SH treatments, after 72 h, were able to statistically reduce the inflammation status, bringing down the threshold to the untreated control ones. No significant difference was revealed between HCC and HCC + SH except for IL-23, where HCC + SH showed a major effect.

#### 2.6.2. Proteins Expression

**Figure 8:**
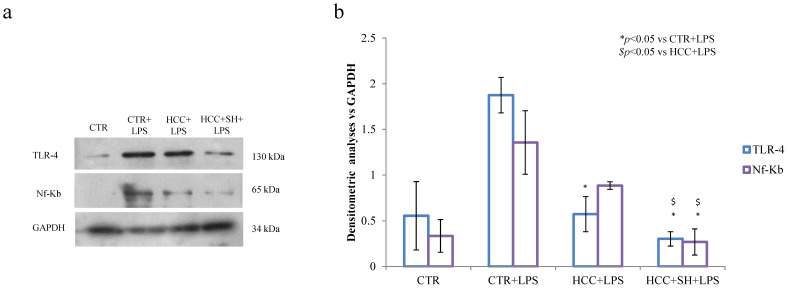
Western blot analysis of NF-κB and TLR-4 in LPS stimulated 3D-skin model at 72 h. GAPDH was used as the loading control. (**a**). Biomarkers involved in inflation process. (**b**). Relative changes of treated samples vs. CTR are reported as mean + SD. * and $ stand for the statistical analyses output *p* < 0.05.

As reported in Figure 8, western blotting analyses showed a significant TLR4 and NF-κB reduction, particularly in presence of HCC + SH respect to LPS-treated control and the treatment with the sole HCC (Supplementary Materials Figure S3).

#### 2.6.3. Inflammatory Cytokines Quantification

**Figure 9:**
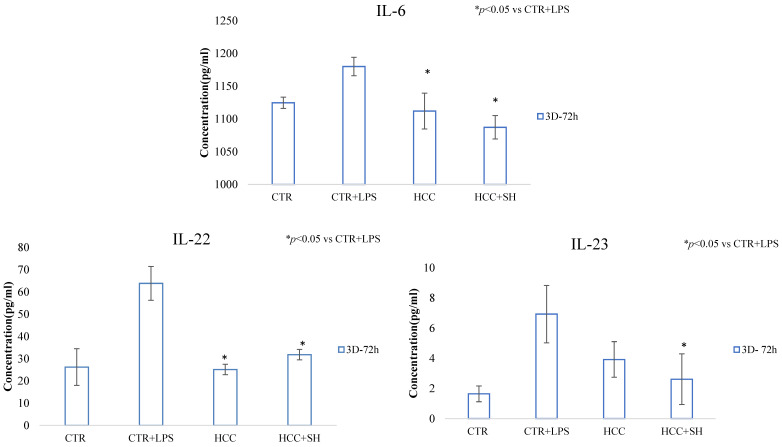
IL-6, IL-22, IL-23 cytokines quantification. * Stand for the statistical analyses output *p* < 0.05.

On injected 3D pieces, cytokines quantification revealed treatments ability to reduce specific inflammation as reported in Figure 9. IL-6 showed a slight increase in presence of LPS, but a statistical decrease was found in presence of HCC and HCC + SH. Regarding IL-22 and IL-23, 1.8 and 1.7 reduction for HCC and 2.6 and 1.6 for HCC + SH, were found respectively.

#### 2.6.4. Immunofluorescence on TLR4

**Figure 10:**
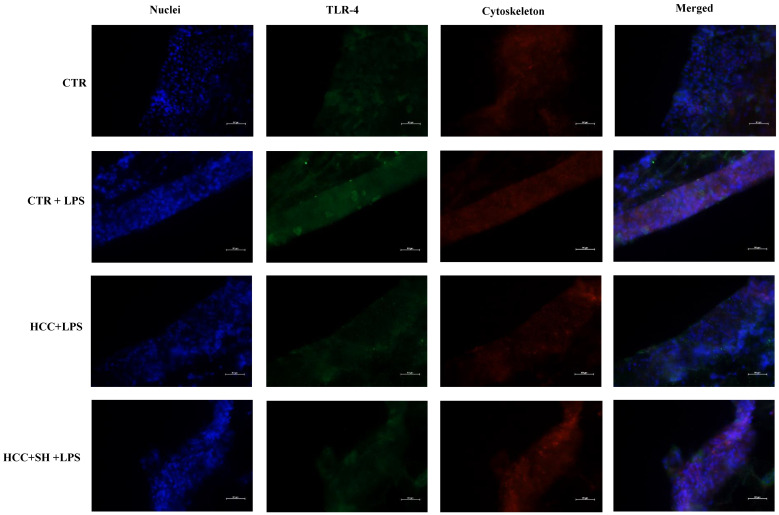
Immunofluorescence TLR-4 staining after 72 h of the treatments on injected 3D skin. Scale bar 100 μm.

Concerning protein levels, immunofluorescence assays indicated a higher expression of TLR-4 (Figure 10) when the 3D skin was stimulated with LPS for 72 h. Treatments showed a marked reduction of the receptor to a level similar to the control, more in presence of HCC + SH (Supplementary Materials Figure S4).

## 3. Discussion

Tissue repair mechanism consists of well-known steps starting from the inflammatory phase up to the cell migration, alongside biosynthesis and assembly of the ECM. In particular, for HCC, the prompting of cell migration, scratch repair and regeneration activities were exploited by TLVM and specific biomarkers evaluation (TGFβ, TNFα, MMPs, RHAMM, collagens and elastin) [3,24]. On the other side, one of the characteristic components of SH hydroalcoholic extract is rosmarinic acid, a polyphenolic compound well known in literature for its efficacy in tissue regeneration and anti-inflammatory role [25,26]. SH was widely studied as protective agent for skin photo aging and senescence [27], and it was recently evaluated in WH process by Scrima et al., confirming this extract as a promising ingredient in skin lesion [25]. Bearing these beneficial properties in mind, the idea to combine these active principles was explored in the present research work. Specifically, results showed that HCC + SH was the formulation that better improved wound closure, corroborating the literature reporting the action each of the components [3,27], also supporting a clinical trial where a novel topical formulation based on HCC and SH was evaluated for its potential benefits in atopic dermatitis patients [11]. In particular, the HCC supplementation to SH extracts prompted positive effects in all in vitro models tested (dehydration, wound healing and inflammation models), and confirmed also its relevance at gene and proteins levels. The experimental data also showed the positive modulation of INTαV and AQP3 in presence of the sole HCC as reported elsewhere [22]. Moreover, treatments with HCC + SH showed a further significant increase for INTαVand for ELS, and, even if with a less remarkable effect, for AQP3. Dehydration test on keratinocytes showed that HCC treated samples counteract desiccation damage, in agreement with previously reported data on a similar model based on corneal cells [28]. For the first time we showed that the presence of SH was able to protect cell monolayer from dehydration. In addition, the efficacy of the coupled formulation (HCC + SH) was superior (*p* < 0.05) compared to the single component treatments. Regarding inflammation models, LPS treatment led to the increase of KRT6 gene expression coherent with a “psoriatic like” HaCaT cells [29]. The presence of treatments, especially for HCC + SH and HCC, showed to counteract this phenomenon, considerably reducing KRT6 gene expression. This result is similar to the effect of daphnetin, reported by Gao and collaborators [18]. No appreciable evidence was found on HaCaT hyperproliferation, as often reported in the literature, but we showed substantial differences between specific genes involved in the inflammation pattern related to psoriasis such as TLR-4, IL-6 and IL-23 [30]. In fact, all these significantly increased after LPS treatment. Generally, TLRs are reported to present expression modulations in psoriasis affected skin when compared to healthy one [31]. In addition, TLR-4 expression increased on peripheral blood mononuclear cells of psoriasis patients compared to controls [32]. Analogously, we found that the presence of LPS increased TLR-4 biosynthesis in all the tested models. In presence of HCC + SH, TLR-4 was statistically reduced at gene and protein level also with respect the sole HCC treatment, confirming the actual efficacy of this formulation suitable to attenuate damages due to PS. IL-6, IL-22 and IL-23 protein expressions were evaluated to be specific PS-related markers. In fact, their sudden increase is reported upon HaCaT treatment with LPS or the so-called M5 (mixture of IL-17A, IL-22, oncostatin M, IL-1α, and TNF-α) resembling the psoriatic skin [18,33]. In our model, these cytokines were reduced in presence of HCC + SH formulations. IL-6 expression was less appreciable similarly to the recent literature [14,34]. It is known that higher IL-23 level promotes the pathogenesis and maintenance of psoriatic lesions and it is one of the primaries signaling pathways leading to characteristic molecular, cellular, and structural changes in PS skin [35]. The latter can be appointed as a key tissue-specific effector cytokine in amplifying the pathology-related inflammatory response [36]. Our data showed that HCC + SH treatment decreased the IL-23 levels about 4-fold, corroborating its ability to counteract psoriatic symptoms. It seems to act even better than Dap, considered as potential anti-psoriatic drug by recent literature [18]. Significant relevance for HCC + SH treatment with respect to the sole HCC was also found for IL-23 gene expression in 3D model. In order to highlight the inflammatory pathway specifically activated in the model resembling PS, we quantified NF-κB protein, that has been shown to be a crucial mediator via TLR-4 [37,38,39,40]. The novel formulations strongly reduced TLR-4 and NF-κB protein expressions in the 3D skin model, as shown for other active components in similar models. The highlighted modulation of the specific biochemical cascade supports the in vivo evidence of the functional role of HCC coupled to SH in commercial preparations [13]. It must be considered that conventional treatments like biologics (e.g., TNF-α inhibitors) increase the risk of systemic immunosuppression, whilst the proposed topical treatment based on HCC + SH exhibit none/minimal side effects [41,42]. This makes the formulation a promising option for long-term management of psoriasis, especially in patients seeking alternatives to systemic treatments.

## 4. Materials and Method

### 4.1. Materials

Hybrid cooperative complexes (HCC) prepared according to the patent reported elsewhere [3] (HHA Mw 1700 ±100 kDa; LHA: Mw = 100 ± 10 kDa) and the dried hydroalcoholic extract of *Salvia haenkei* (SH-Haenkenium^®^), (batch: PPO004591/710) were gifted by IBSA Farmaceutici Italia Srl (Lodi, Italy). A specific amount of the dried SH was dissolved in dimethyl sulfoxide (DMSO) at final concentration of 10 mg/mL. SH was diluted 1:1000 in the medium to always have 0.1% *v*/*v* of DMSO suitable for cell viability [12]. Lipopolysaccharides (LPS; 5 × 106 EU of VacciGrade™ lipopolysaccharide from E. coli 0111: B4; LPS-EB VacciGrade™) was purchased by InvivoGen (Toulouse, France) and it was diluted at a final concentration of 1 and 2 μg/mL in culture medium for proliferation assay [17] and 3D skin model [43] respectively. Human keratinocytes (HaCaT) were provided by Zooprofilattico institute of Brescia (Brescia, Italy), and the cells were cultured in Dulbecco’s modified Eagle medium (DMEM), supplemented as described elsewhere. Phenion^®^ Full Thickness Skin Model (FT-skin), produced by Henkel (Dusseldorf, Germany, diameter 1.3 cm) was used for the 3D-experiments, as previously described elsewhere [7].

### 4.2. Methods

#### 4.2.1. Dehydration Test

The dehydration test was accomplished using HaCaT cell lines, growth in a standard 24-well culture plate, until a confluence of 50–60%, to observe better cell morphology. The cells were treated for 2 h with SH (10 μg/mL), HCC (0.2% *w*/*w*) and HCC formulation supplemented with SH (respectively 0.2% *w*/*w* + 10 μg/mL). After 2 h, the treatments were removed (except for the positive control, CTR+, where DMEM 10% FBS was left for all the experiment) and the plate was then left open in the incubator (at 37 °C and 5%CO_2_ in humidified air) for 20 min to mimic a stress condition, as reported in La Gatta et al. [28]. The dehydrated control (CTR−) was processed according to the protocol but without adding any treatment. Cells viability was evaluated by 3-(4,5-dimethylthiazol-2-yl)-2,5-diphenyl-2H-tetrazolium bromide (MTT) assay, as described elsewhere [6]. An optical microscope was used to record the cells prior to the dehydration test and their shape alteration after the suffering status.

#### 4.2.2. In Vitro Keratinocytes Scratch Test and Time Lapse Video Microscopy (TLVM)

To evaluate the biological properties of formulations on wound closure (HCC, SH and HCC + SH) HaCaT cells monolayer was used as simplified model of dermal human skin. The injury was created (approximately 0.5–0.9 mm in width) mechanically with a sterile pipette tip (Ø = 0.1 mm) and washed with PBS to remove cellular debris. The scratched monolayers were incubated for overall the experiment with HCC 2 mg/mL, SH 10 μg/mL and the mixture of HCC + SH (at same concentration) diluted in DMEM 1% FBS. The control is diluted similarly to the samples using PBS. The in vitro wound healing assay was performed by video microscopy time lapse experiments (TLVM) (OKOLAB, Pozzuoli, Italy), as described elsewhere [6].

#### 4.2.3. Proliferation Assay in 2D Model

HaCaT cells proliferation was monitored at different times (24, 48 and 72 h) through time lapse experiment measuring the increase of occupied area of cells respect to the one at time zero and eventually observing the anomalous cell growth due to LPS addition. In more detail, 3 × 104 cells were seeded on 24-wells and after cell adhesion (3–4 h) the treatments (HCC; HCC + SH; SH) were added in each well. The experiment was run in triplicates. After 24 h, the medium containing the formulations was removed and fresh medium containing LPS (1 μg/mL) was added. The positive (CTR) and negative control (CTR + LPS) were respectively treated without and with LPS [18].

#### 4.2.4. In Vitro Inflammation in 2D and 3D Models

In the 2D, HaCaT cells were treated with LPS, an inflammatory agent, normally used in psoriatic model as described elsewhere [18]. The sole medium without LPS represented the control and confirmed that the inflammation occurred. For 3D model FT-skin, HCC and HCC + SH gels were injected in six different points (50 μL each) of the FT-skin specimen just below the epidermal surface. The samples were then incubated for 72 h in medium containing LPS (2 μg/mL). Control samples were injected with PBS solution (6 injections of 50 μL). FT-skin portions were collected and treated according to the manufacturer ‘instructions for gene and protein expressions.

#### 4.2.5. qRT-PCR Analyses on HaCaT and 3D-Skin

Inflammatory biomarkers were evaluated at 24 h and 72 h for 2D and 3D respectively. They were lysed with TRIzol^®^ (Invitrogen, Milan, Italy) and after quantification of extracted RNA, through a Nanodrop spectrophotometer (Celbio, Milan, Italy), total RNA was transcribed in the cDNA using Reverse Transcription System Kit (Promega, Milan, Italy). The cDNA was mixed with iQ™ SYBR^®^ Green Supermix (Bio-Rad Laboratories Srl, Milan, Italy) and primer solutions for the target gene KRT6, TLR4, IL6, IL23 (Table 1, designed by Beacon Designer™ software 7.9) for quantitative real time PCR (qRT-PCR). Samples were run in duplicate and the expression of specific mRNA relative to the control was determined after normalization with respect to hypoxanthine guanine phosphoribosyl transferase (GAPDH) housekeeping gene (internal control) [44].

#### 4.2.6. Western Blotting Analysis on HaCaT Monolayer Scratched or Not and on 3D-Skin

Western blot analyses were performed on scratched HaCaT cells and 3D skin model, at the end of experiments (72 h), to evaluate respectively reepithelization and inflammations biomarkers. RIPA buffer (composed by 20 mM Tris-HCl, 150 mM NaCl, 1 mM Na_2_EDTA, 1 mM EGTA, 1% NP-40, 1% sodium deoxycholate, 2.5 mM sodium pyrophosphate, 1 mM b-glycerophosphate, 1 mM Na_3_VO_4_ and 1 μg/mL leupeptin) was used to lysate the cells (Cell Signaling Technology, Danvers, MA, USA), and Bradford method to determine proteins concentration [45]. Successively, 20 μg of cells lysate were loaded and run using 10% SDS–PAGE. The separated proteins were then transferred to nitrocellulose membrane (Amersham) and blocked in 5% milk solution. INTαV (125 kDa), Elastin (60 kDa) and AQP3 (60 kDa) were utilized to evaluate remodelling and TLR4 (130 kDa) and NF-κB (65 kDa) for inflammation (diluted 1:250 in BSA solution). Immunoreactive bands were detected by chemiluminescence using corresponding secondary antibody (1:3000 dilution). The membranes were then reacted with an ECL system (Chemicon-Millipore, Milan, Italy) and protein levels were normalized with respect to GAPDH housekeeping protein (1:1000 dilutions). The semi-quantitative analysis of protein levels was carried out by Image J (version 1.54 g).

#### 4.2.7. IL-6, IL-22 and IL-23 Quantification by ELISA

Supernatants of HaCaT proliferation cell cultures (24, 48 and 72 h) and 3D skin model (only 72 h) challenged with LPS, were analysed to quantify IL-6, IL-22 and IL-23 productions using ELISA assay following the manufacturer’s suggested protocol (Boster antibody and ELISA experts, Tema Ricerca, Padova, Italy). Cells supernatants were collected and centrifuged (3000 rpm for 10 min at 4 °C) and then transferred to a microtiter plate for incubation with specific biotinylated polyclonal antibody for 1 h. Each experiment was performed in triplicate and optical densities were measured at 450 nm using a microplate reader (Biorad laboratories., Segrate, Italy). The concentrations were calculated using a standard curve according to the manufacturer’s instructions.

#### 4.2.8. TLR4 Immunofluorescence Staining on 3D-Skin Sections

TLR4 protein level was detected on FT-skin tissues by immunofluorescence (IF) staining as reported elsewhere [5]. Briefly, FT-skin sections, injected and incubated for 72 h with the above-mentioned formulations were fixed in a Paraformaldehyde (PFA) solution for three days at 4 °C after that they were left in 30% sucrose solution for 1 week. Then included in cryostat embedding medium (Bioptica, Milan, Italy) to be cryo-sectioning. For antigen retrieval the sections, collected on slides, were incubated for 10 min at 95 °C in tris-EDTA buffer (10 mM Tris base, 1 mM EDTA solution, 0.05% Tween, pH 9). To permeabilize samples, blocking solution was added in a humid chamber. For antibody detection Primary antibodies against TLR4 (specific inflammation biomarker) (diluted 1:100; Abcam, Cambridge, MA, UK) were incubated over-night. Afterwards, the slices were washed in PBS and further incubated with FITC-conjugated goat anti-rabbit secondary antibodies (diluted 1:1000; Life Technologies, Milano, Italy) for 45 min. Cellular nuclei were stained with 10 μL DAPI (Mounting medium with DAPI; Abcam, Abcam, Cambridge, MA, UK). Finally, the slices were sealed for observation; specific images were obtained by a fluorescence microscope Nikon Eclipse Ci (Melville, NY, USA) equipped with software Nis-Elements 4.4000.00.

#### 4.2.9. Statistical Analyses

Each experiment was performed at least three times. The results were expressed as mean ± standard deviations (SD). The significant differences among the groups were assessed using Student’s t-test. Significant differences were marked in figure (e.g., *, &, §, $, £ etc.) for *p* values lower than 0.05.

## 5. Conclusions

In the framework of this research, HCC + SH formulations were fully characterized, in comparison to the single components, showing to promote tissue regeneration, wound healing and counteract dehydration. ELS, INTαV and AQP3 protein expression confirmed a sound tissue formation. Generally, in all the diverse in vitro inflammation models, resembling PS, HCC and HCC + SH treatments led to a reduction of inflammatory signals in terms of TLR-4, NF-κB and IL-6, IL-23 expression level, also confirmed in 3D model dermal model. The synergistic effect of HCC + SH suggests that this combination could be more effective than existing psoriasis treatments that focus solely on managing symptoms without aiding skin regeneration.

## Figures and Tables

**Table 1:** Primer sequences for specific biomarkers employed for the qRT-PCR.

Gene Name	Primer Sequence (5′–3′)	AT PCR
*GAPDH*	FORWARD 5′-TGCACCACCAACTGCTTAGC-3′ REVERSE 5′-GGCATGGACTGTGGTCATGAG-3′	55 °C
*TLR-4*	FORWARD 5′-AAGCCGAAAGGTGATTGTTG-3′ REVERSE 5′-CTGAGCAGGGTCTTCTCCAC-3′	60 °C
*KRT-6*	FORWARD 5′-GGGTTTCAGTGCCAACTCAG-3′ REVERSE 5′-CCAGGCCATACAGACTGCGG-3′	60 °C
*IL-6*	FORWARD 5′-GTACTCTAGACCAGAGG-3′ REVERSE 5′-TGCTGGTGACAACCACGGCC-3′	55 °C
*IL-23*	FORWARD 5′-CTCAGGGACAACAGTCAGTTC-3′ REVERSE 5′-ACAGGGCTATCAGGGAGCA-3′	52 °C

## Data Availability

All data are reported in the manuscript, graphs and table. Row data are available on request to the correspondent author.

## Supplementary Materials

The following supporting information can be downloaded at: https://www.mdpi.com/article/10.3390/ijms26031310/s1.
