# Supplementary material for: Protective and Anti-Inflammatory Effect of Novel Formulation Based on High and Low Molecular Weight Hyaluronic Acid and Salvia haenkei
Source: Int J Mol Sci. 2025 Feb 4;26(3):1310. doi: 10.3390/ijms26031310 (PMC11818062; doi:10.3390/ijms26031310)
Supplement: Supplementary file 1 [file ijms-26-01310-s001.zip › ijms-3367904 supplementary renumbered.docx]

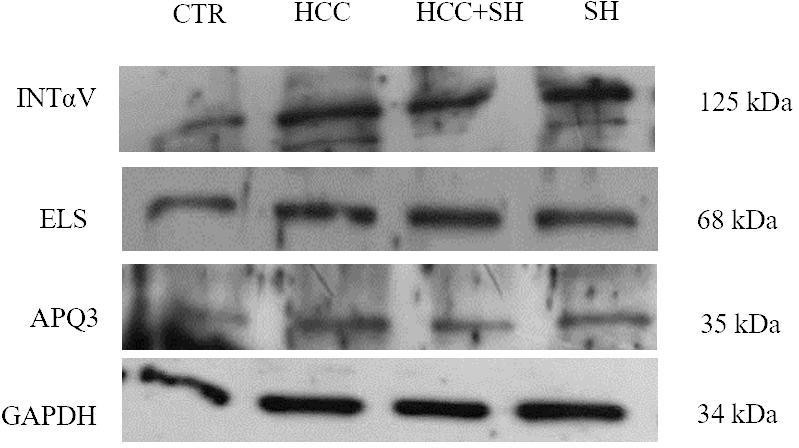


Figure S1: Second western blot analysis of INTαV, ELS and AQP3 in scratched HaCaT monolayer at 72h in the CTR and in presence of different treatments


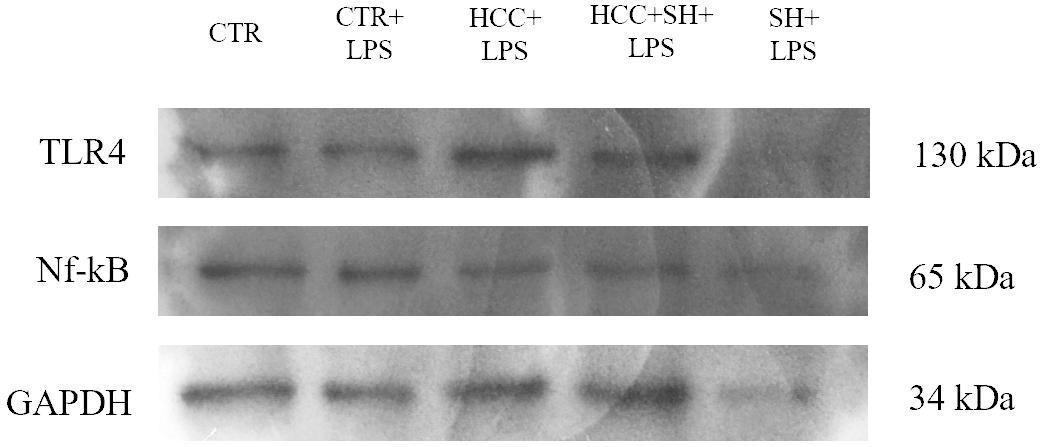


Figure S2: Second Western blot analysis in inflamed HaCaT cell at 72h. GAPDH was used as the loading control


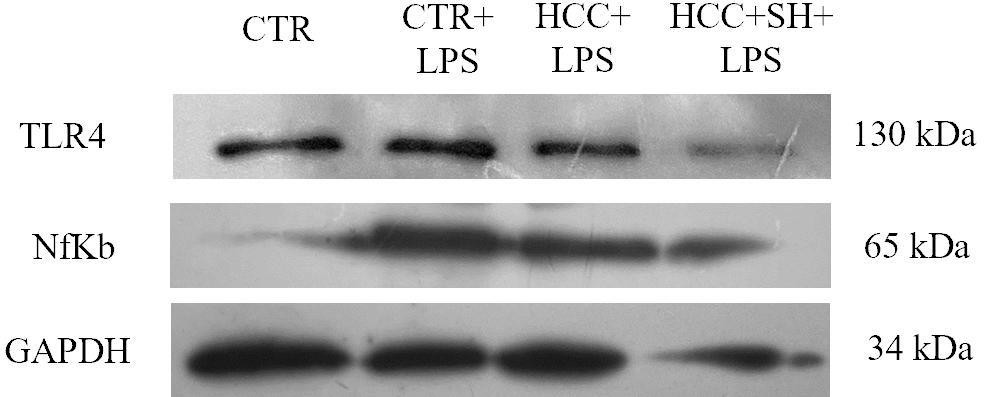


Figure S3: Second Western blot analysis in LPS stimulated 3D- skin model at 72h. GAPDH was used as the loading control


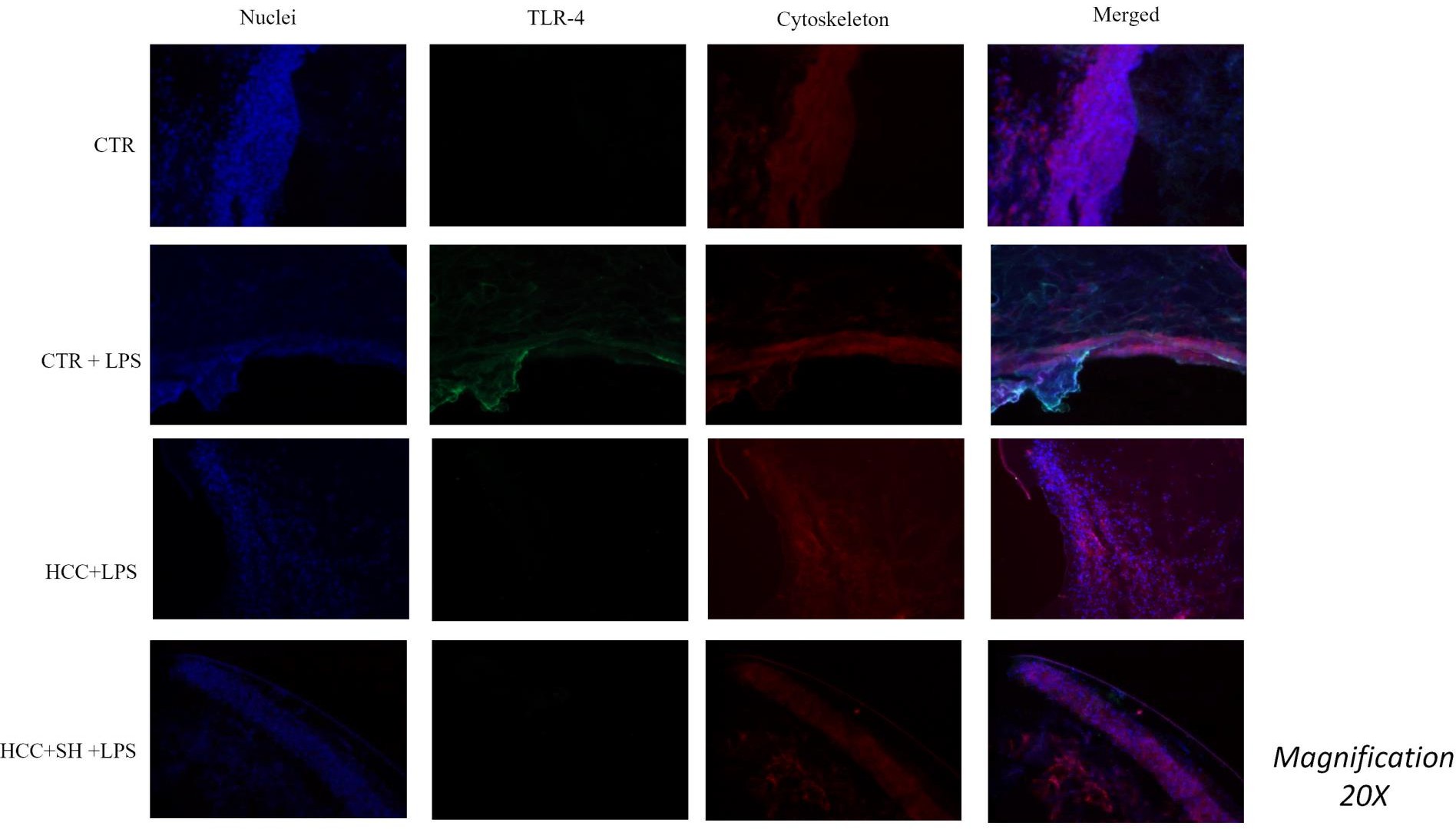


Figure S4: Second panel of immunofluorescence TLR-4 staining after 72h of the treatments on injected 3D skin.
